# The Influence of Nonlinear High-Intensity Dynamic Processes on the Standing Wave Precession of a Non-Ideal Hemispherical Resonator

**DOI:** 10.3390/s24092709

**Published:** 2024-04-24

**Authors:** Wei Cheng, Shunqing Ren, Boqi Xi, Zhen Tian, Youhuan Ning, Yan Huo

**Affiliations:** Space Control and Inertial Technology Research Center, Harbin Institute of Technology, Harbin 150080, China; 21b904034@stu.hit.edu.cn (W.C.); renshunqing@hit.edu.cn (S.R.); 22b904048@stu.hit.edu.cn (Z.T.); 20b904047@stu.hit.edu.cn (Y.N.); yhuo@hit.edu.cn (Y.H.)

**Keywords:** hemispherical resonator, dynamic model, frequency splitting, nonlinearity, method of averaging

## Abstract

The properties of small size, low noise, high performance and no wear-out have made the hemispherical resonator gyroscope a good choice for high-value space missions. To enhance the precision of the hemispherical resonator gyroscope for use in tasks with large angular velocities and angular accelerations, this paper investigates the standing wave precession of a non-ideal hemispherical resonator under nonlinear high-intensity dynamic conditions. Based on the thin shell theory of elasticity, a dynamic model of a hemispherical resonator is established by using Lagrange’s second kind equation. Then, the dynamic model is equivalently transformed into a simple harmonic vibration model of a point mass in two-dimensional space, which is analyzed using a method of averaging that separates the slow variables from the fast variables. The results reveal that taking the nonlinear terms about the square of the angular velocity and the angular acceleration in the dynamic equation into account can weaken the influence of the 4th harmonic component of a mass defect on standing wave drift, and the extent of this weakening effect varies with the dimensions of the mass defects, which is very important for steering the development of the high-precision hemispherical resonator gyroscope.

## 1. Introduction

The hemispherical resonator gyroscope (HRG) is a classic Coriolis vibration gyroscope [1,2,3,4], which utilizes the Coriolis effect on standing waves in an axisymmetric shell to measure angular velocity with respect to the inertial space. Its characteristics of high dynamics, high precision, high reliability, long service life, low power consumption and miniaturization have attracted wide attention in recent years [5,6,7,8,9,10,11].

The basic fundamentals utilized in the HRG were discovered over a century ago by G.H. Bryan [12]. The hemispherical shell, also known as the hemispherical resonator, is the core component of the HRG. During the production of a hemispherical resonator, an uneven distribution of mass can occur due to a workpiece clamping eccentricity, tool vibration, or uneven tool wear, any of which will cause a decrease in the performance of the gyroscope. There is a large amount of literature about mass defects in resonators [13,14,15,16,17,18]. However, when studying dynamic models, researchers often use simple linear models, and few pay attention to the effects of nonlinearity. In reference [7], the geometrical and electrical nonlinearities of the hemispherical resonator were taken into account and the equation of motion for the resonator was analyzed under parametric excitation. The nonlinear dynamic equation for a rotating elastic ring resonator was established and solved by a numerical-analytical method based on both the generalized Bubnov–Galerkin method (Kantorovich method) and the direct method (Rothe method) [19]. In addition, with a linear law of the angular rate, a nonlinear dynamic equation for the Coriolis vibratory gyroscope was established, and the solution was expressed in terms of the Weber functions (the parabolic cylinder functions) [20]. Due to the complexity of the nonlinear models, in reference [21] a precise low-order model was studied by perturbation analysis with the Galerkin method (or Ritz method), accounting for modal coupling and interactions. In reference [22], which discussed the von Karman nonlinearity, a high-dimensional nonlinear dynamical equation for the shell of revolution was formulated based on Love’s theory, and the influence of meridian geometry on natural frequency was studied. The above references are crucial resources for researching nonlinear dynamic models of hemispherical resonators. However, they mainly focused on methods for solving natural frequencies or nonlinear equations, without studying the influence of nonlinearity on standing wave precession.

In this paper, a dynamic model of a hemispherical resonator is established by considering the mass defects of the resonator and high-intensity dynamic processes. The dynamic model is equivalent to a point mass harmonic vibration model in a two-dimensional space. The equivalent model is analyzed using a method of averaging that separates the slow variables from the fast variables to investigate the influence of nonlinear high-intensity dynamic processes on the standing wave precession of a non-ideal hemispherical resonator. The results reveal that taking into account the nonlinear terms about the square of the angular velocity and the angular acceleration in the dynamic equation can weaken the influence of the 4th harmonic component of the mass defect on standing wave drift. The extent of this weakening effect varies with the dimensions of the mass defects.

## 2. Methods

### 2.1. Basic Structure and Working Principle of HRG

**Figure 1:**
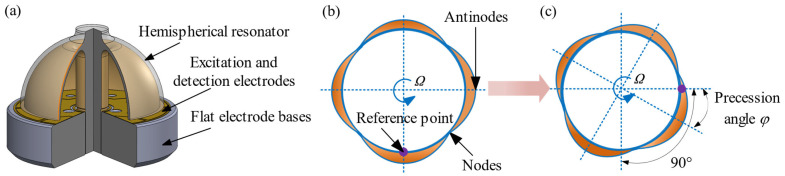
(**a**) Structure diagram of the hemispherical resonator gyroscope; (**b**) Operation mode shape of the hemispherical resonator; (**c**) Precession effect of the standing wave.

A hemispherical resonator gyroscope utilizes the rotation sensitivity of the second order vibration mode of a hemispherical shell to measure the rotational angular velocity or rotational angle of a carrier. This hemispherical shell is called a hemispherical resonator, as shown in Figure 1a. When the resonator is vibrating in the second order mode, the vibrating equatorial ellipse forms a standing wave with four equally-spaced antinodes (locations of maximum displacement) and four nodes (locations of zero displacement, at least in the radial direction) in between [5], as shown in Figure 1b. When such a standing wave is present in the shell, a rotation about the shell axis (the symmetric axis of the stem) produces Coriolis forces on the vibrating mass elements, causing the standing wave to change its position on the shell, as shown in Figure 1c.

Ideally, the relationship between the precession angle *φ* of the standing wave and the angular velocity *Ω* is $\dot{\varphi }$ = *KΩ*. *K* is called the precession coefficient, also known as the Bryan factor [5,12].

### 2.2. Dynamical Models of Hemispherical Resonators

The hemispherical resonator is the core component of a hemispherical resonator gyroscope, and its vibration characteristics are critical to the performance of the gyroscope. In order to describe the deformation and motion of the hemispherical resonator, dynamic models of an ideal and non-ideal hemispherical resonator are established based on the thin shell theory of elasticity and the Lagrange’s second kind equation.

#### 2.2.1. Strain Energy of Hemispherical Resonator

**Figure 2:**
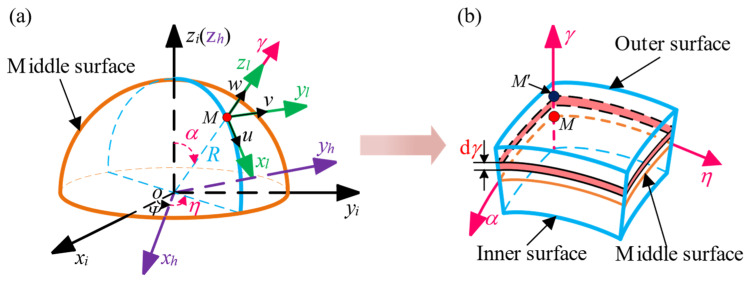
(**a**) Related coordinate systems; (**b**) Micro-element body of a hemispherical resonator.

The thickness of a hemispherical resonator is much smaller than its surface radius, based on the thin shell theory of elasticity [23]. The geometric equation of the hemispherical resonator can be expressed as

(1) $$\left\{\begin{array}{l} \varepsilon_{1}=\frac{1}{R}(\frac{\partial u}{\partial \alpha }+w),\\ \varepsilon_{2}=\frac{1}{R\sin\alpha }(\frac{\partial v}{\partial \eta }+u\cos\alpha +w\sin\alpha ),\\ \varepsilon_{12}=\frac{1}{R\sin\alpha }(\frac{\partial u}{\partial \eta }+\frac{\partial v}{\partial \alpha }\sin\alpha -v\cos\alpha ), \end{array}\right.\;\;\left\{\begin{array}{l} \chi_{1}=-\frac{1}{R^{2}}\frac{\partial^{2}w}{\partial \alpha^{2}}-\frac{w}{R^{2}},\\ \chi_{2}=-\frac{1}{R^{2}\sin^{2}\alpha }\frac{\partial^{2}w}{\partial \eta^{2}}-\frac{\cot\alpha }{R^{2}}\frac{\partial w}{\partial \alpha }-\frac{w}{R^{2}},\\ \chi_{12}=-\frac{1}{R^{2}\sin\alpha }(\frac{\partial^{2}w}{\partial \alpha \partial \eta }-\cot\alpha \frac{\partial w}{\partial \eta }), \end{array}\right.$$

where *ε*_1_ and *ε*_2_ are the normal strains of point *M* on the middle surface in the directions *α* and *η* respectively. *ε*_12_ is the shear strain. *χ*_1_ and *χ*_2_ are the changes in the principal curvature. *χ*_12_ is the change in the twist rate in the directions *α* and *η*, as shown in Figure 2. *R* is the radius of the middle surface. The displacement of point *M* is expressed by *u*, *v* and *w*, respectively, after the hemispherical resonator is deformed.

The strain relationships between point *M*′ and corresponding point *M* are

(2) $$e_{1}=\varepsilon_{1}+\chi_{1}\gamma ,\;e_{2}=\varepsilon_{2}+\chi_{2}\gamma ,\;e_{12}=\varepsilon_{12}+2\chi_{12}\gamma ,$$

where the normal strains of point *M*′ in the directions *α* and *η* are expressed by *e*_1_ and *e*_2_ and the shear strain is expressed by *e*_12_, as shown in Figure 2. Based on the thin shell theory of elasticity [23], the physical equation of the hemispherical resonator can be expressed as

(3) $$\left\{\begin{array}{l} \sigma_{1}=\frac{E}{1-\mu^{2}}[(\varepsilon_{1}+\mu \varepsilon_{2})+(\chi_{1}+\mu \chi_{2})\gamma ],\\ \sigma_{2}=\frac{E}{1-\mu^{2}}[(\varepsilon_{2}+\mu \varepsilon_{1})+(\chi_{2}+\mu \chi_{1})\gamma ],\\ \tau_{12}=\frac{E}{2(1+\mu )}(\varepsilon_{12}+2\chi_{12}\gamma ), \end{array}\right.$$

where the normal stresses at point *M*′ in the directions *α* and *η* are expressed by *σ*_1_ and *σ*_2_, respectively. Shear stress is expressed by *τ*_12_. *μ* is Poisson’s ratio and *E* is Young’s modulus.

Then, the strain energy of the hemispherical resonator is

(4) $$E_{P}=\frac{1}{2}R^{2}\int_{-\frac{h}{2}}^{\frac{h}{2}}\int_{0}^{2\pi }\int_{0}^{\frac{\pi }{2}}\left(\sigma_{1}e_{1}+\sigma_{2}e_{2}+\tau_{12}e_{12}\right)\sin\alpha \;\;d\alpha d\eta d\gamma .$$

Substituting Equations (1)–(3) into Equation (4), the strain energy is simplified as

(5) $$E_{p}=b_{0}(p^{2}+q^{2}),$$

where the coefficient *b*_0_ is a constant value after integration, which can be found in Appendix A, Equation (A1). Here, the mass defect does not affect the potential energy. Next, the kinetic energy will be calculated separately for the ideal and non-ideal resonators.

#### 2.2.2. Kinetic Energy of the Hemispherical Resonator

In order to reflect the mass defects of the non-ideal hemispherical resonator, we assume that its thickness is uniform, and we expand its density into a Fourier series with respect to the circular angle. When the hemispherical resonator operates in the second order vibration mode, the displacement of point *M* on the middle surface is [18,24]

(6) $$\left\{\begin{array}{l} u(\alpha ,\eta ,t)=U(\alpha )[p(t)\cos2\eta +q(t)\sin2\eta ],\\ v(\alpha ,\eta ,t)=V(\alpha )[p(t)\sin2\eta -q(t)\cos2\eta ],\\ w(\alpha ,\eta ,t)=W(\alpha )[p(t)\cos2\eta +q(t)\sin2\eta ], \end{array}\right.$$

where *p*(*t*) and *q*(*t*) are the generalized coordinates, reflecting the changes in two modes of the hemispherical resonator over time. *U*(*α*), *V*(*α*) and *W*(*α*) are the Rayleigh functions [24,25], as follows.

(7) $$\left\{\begin{array}{l} U(\alpha )=\sin\alpha \tan^{2}(\frac{\alpha }{2}),\\ V(\alpha )=-\sin\alpha \tan^{2}(\frac{\alpha }{2}),\\ W(\alpha )=-(2+\cos\alpha )\tan^{2}(\frac{\alpha }{2}). \end{array}\right.$$

When the hemispherical resonator rotates around its axis of rotational symmetry in inertial space at any angular velocity *Ω*, the absolute speed ***V**_a_* of point *M*′ in the shell is

(8) $$V_{a}=\left[\begin{array}{c} -\Omega [v(1+\frac{\gamma }{R})-\frac{\gamma }{R\sin\alpha }\frac{\partial w}{\partial \eta }]\cos\alpha +\dot{u}(1+\frac{\gamma }{R})-\frac{\gamma }{R}\frac{\partial \dot{w}}{\partial \alpha }\\ \Omega (R+\gamma +w)\sin\alpha +\Omega u\cos\alpha +\dot{v}(1+\frac{\gamma }{R})-\frac{\gamma }{R\sin\alpha }\frac{\partial \dot{w}}{\partial \eta }\\ -\Omega [v(1+\frac{\gamma }{R})-\frac{\gamma }{R\sin\alpha }\frac{\partial w}{\partial \eta }]\sin\alpha +\dot{w} \end{array}\right],$$

Then, the kinetic energy of the hemispherical resonator is

(9) $$E_{T}=\frac{1}{2}R^{2}\int_{-\frac{h}{2}}^{\frac{h}{2}}\int_{0}^{2\pi }\int_{0}^{\frac{\pi }{2}}{\left(v_{a}\right)}^{2}\rho \sin\alpha \;d\alpha d\eta d\gamma .$$

The thickness *h* and density *ρ* of the ideal hemispherical resonator are uniform and constant. Substituting Equations (6)–(8) into Equation (9), the kinetic energy of the ideal hemispherical resonator is

(10) $$E_{T}=a_{0}\Omega^{2}+a_{1}({\dot{p}}^{2}+{\dot{q}}^{2})+a_{2}(q\dot{p}-p\dot{q})\Omega +a_{3}(p^{2}+q^{2})\Omega^{2},$$

where coefficients *a*_0_, *a*_1_, *a*_2_ and *a*_3_ are all constant values after integration. The expressions for them are complicated and cumbersome, but they do not affect the following theoretical analysis, so the specific integral formulas of these coefficients are not listed here. The integral formulas can be found in Appendix A, Equation (A2).

In order to reflect a mass defect in a non-ideal hemispherical resonator, the density *ρ* is expanded into a Fourier series with respect to the circular angle *η*. Since the frequency splitting of the hemispherical resonator is mainly caused by the 4th harmonic term [18], only the constant term and the 4th harmonic term are retained here.

(11) $$\rho (\eta )=\rho_{0}[1+\varepsilon_{4}\cos(4(\eta -\theta_{4}))],$$

where $\rho_{0}$ is the average density (equal to the density *ρ* of the ideal hemispherical resonator) and *ε*_4_ and *θ*_4_ are the relative amplitude and phase angle of the 4th harmonic of density anisotropy, respectively.

Substituting Equations (6)–(8) and (11) into Equation (9), the kinetic energy of the non-ideal hemispherical resonator is

(12) $${\bar{E}}_{T}={\bar{a}}_{0}\Omega^{2}+{\bar{a}}_{1}{\dot{p}}^{2}+{\bar{a}}_{2}{\dot{q}}^{2}+({\bar{a}}_{3}q\dot{p}-{\bar{a}}_{4}p\dot{q})\Omega +({\bar{a}}_{5}p^{2}+{\bar{a}}_{6}q^{2}+{\bar{a}}_{7}pq)\Omega^{2}+{\bar{a}}_{8}\dot{p}\dot{q},$$

where ${\bar{a}}_{0}\cdots {\bar{a}}_{8}$ are all constant values after integration. Similarly, the expressions for them are not listed here, but can be found in Appendix A, Equation (A3).

#### 2.2.3. Dynamic Equations of the Hemispherical Resonators

The dynamic equations of an ideal and non-ideal resonator are established by the Lagrange’s second kind equation using the kinetic energy and strain energy of the resonators.

(13) $$\left\{\begin{array}{l} \frac{d}{dt}(\frac{\partial L}{\partial \dot{p}})-\frac{\partial L}{\partial p}=0,\\ \;\frac{d}{dt}(\frac{\partial L}{\partial \dot{q}})-\frac{\partial L}{\partial q}=0, \end{array}\right.$$

where the expression of the Lagrange function *L* is *L* = *E_T_* − *E_p_*. Substituting Equations (5) and (10) into Equation (13), the dynamic equation of the ideal hemispherical resonator is

(14) $$\left\{\begin{array}{l} \ddot{p}+c_{1}\Omega \dot{q}+(c_{3}-c_{2}\Omega^{2})p+\frac{c_{1}}{2}\dot{\Omega }q=0,\\ \ddot{q}-c_{1}\Omega \dot{p}+(c_{3}-c_{2}\Omega^{2})q-\frac{c_{1}}{2}\dot{\Omega }p=0, \end{array}\right.$$

where $c_{1}=\frac{a_{2}}{a_{1}},c_{2}=\frac{a_{3}}{a_{1}},c_{3}=\frac{b_{0}}{a_{1}}.$

The natural frequency of the ideal hemispherical resonator is

(15) $$\;f=\frac{\omega }{2\pi }=\frac{\sqrt{c_{3}}}{2\pi }.$$

Substituting Equations (5) and (12) into Equation (13), the dynamic equation of the non-ideal hemispherical resonator is

(16) $$\left\{\begin{array}{l} \ddot{p}+\frac{c_{11}+c_{12}}{2}\Omega \dot{q}+(c_{16}-c_{13}\Omega^{2})p+(\frac{c_{11}}{2}\dot{\Omega }-\frac{c_{14}}{2}\Omega^{2})q+\frac{c_{15}}{2}\ddot{q}=0,\\ \ddot{q}-\frac{c_{21}+c_{22}}{2}\Omega \dot{p}+(c_{26}-c_{23}\Omega^{2})q+(-\frac{c_{22}}{2}\dot{\Omega }-\frac{c_{24}}{2}\Omega^{2})p+\frac{c_{25}}{2}\ddot{p}=0, \end{array}\right.$$

where

(17) $$\begin{array}{l} c_{11}=\frac{{\bar{a}}_{3}}{{\bar{a}}_{1}},\;c_{12}=\frac{{\bar{a}}_{4}}{{\bar{a}}_{1}},\;c_{13}=\frac{{\bar{a}}_{5}}{{\bar{a}}_{1}},\;c_{14}=\frac{{\bar{a}}_{7}}{{\bar{a}}_{1}},\;c_{15}=\frac{{\bar{a}}_{8}}{{\bar{a}}_{1}},\;c_{16}=\frac{b_{0}}{{\bar{a}}_{1}},\;\\ c_{21}=\frac{{\bar{a}}_{3}}{{\bar{a}}_{2}},\;c_{22}=\frac{{\bar{a}}_{4}}{{\bar{a}}_{2}},\;c_{23}=\frac{{\bar{a}}_{6}}{{\bar{a}}_{2}},\;c_{24}=\frac{{\bar{a}}_{7}}{{\bar{a}}_{2}},\;c_{25}=\frac{{\bar{a}}_{8}}{{\bar{a}}_{2}},\;c_{26}=\frac{b_{0}}{{\bar{a}}_{2}}. \end{array}$$

The natural frequencies of the two modes are no longer equal due to the mass defect.

(18) $$\omega_{1}=\sqrt{c_{16}},\;\omega_{2}=\sqrt{c_{26}}.$$

Therefore, the frequency splitting equation is

(19) $$\Delta f=\frac{\Delta \omega }{2\pi }=\frac{\omega_{1}-\omega_{2}}{2\pi }.$$

In this paper, frequency splitting is only used to reflect the dimensions of mass defects which, when calculated with no angular velocity *Ω*, can effectively reflect the dimensions of mass defects. In addition, we mainly focus on the precession of the standing wave in this paper, but centrifugal force does not cause the precession of the standing wave [26]. To simplify the analysis, we do not consider the effects of centrifugal force in Equations (15), (18) and (19). Equation (16) is the starting point for analyzing the influence of nonlinear high-intensity dynamic processes on standing wave precession in a non-ideal hemispherical resonator.

### 2.3. Method of Averaging

Since the generalized coordinates *p* and *q* change very rapidly with time (they are called the fast variables), it is difficult to establish a clear correlation with the angular velocity. However, the parameters of the ellipse determined by the trajectory of a point (*p*, *q*) change very slowly. These parameters are called the slow variables and can be well correlated to the angular velocity. The dynamic models of the ideal and non-ideal hemispherical resonators are solved using the method of averaging, separating the slow variables from the fast variables.

#### 2.3.1. Solution to the Ideal Hemispherical Resonator

To facilitate the following analysis, the dynamic equation of the ideal hemispherical resonator is simplified into the following form.

(20) $$\left\{\begin{array}{l} \ddot{p}+\omega^{2}p=-k_{1}\Omega \dot{q}-k_{2}\dot{\Omega }q+k_{3}\Omega^{2}p,\\ \ddot{q}+\omega^{2}q=k_{1}\Omega \dot{p}+k_{2}\dot{\Omega }p+k_{3}\Omega^{2}q, \end{array}\right.$$

where *p* and *q* are the generalized coordinates, that is, the fast variables we mentioned at the beginning. In addition, $\omega^{2}=c_{3},\;\;k_{1}=c_{1}\;,\;\;k_{2}=\frac{1}{2}c_{1},\;\;k_{3}=c_{2}.$

**Figure 3:**
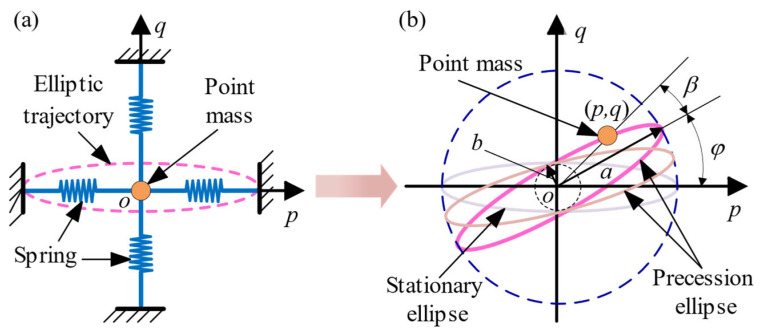
(**a**) Vibration model of a point mass in a two-dimensional space; (**b**) Elliptic trajectory of the point (*p*, *q*).

When there is no angular velocity acting on the resonator, the solution to Equation (20) has the following form [27]

(21) $$\left\{\begin{array}{l} p=a\cos\theta \cos\varphi -b\sin\theta \sin\varphi ,\\ q=a\cos\theta \sin\varphi +b\sin\theta \cos\varphi , \end{array}\right.$$

where θ=ωt+β. Then, Equation (21) is equivalent to the linear vibration equation of a point mass in the two-dimensional space, as shown in Figure 3a. The point (*p*, *q*) represents the generalized displacement of the point mass [27], and its motion trajectory is an ellipse.

If the angular velocity is not zero, then parameters *a*, *b*, *φ* and *β* (these four parameters are called the slow variables) will change over time. Parameter *a* represents the major semi-axis of the ellipse, *b* represents the minor semi-axis of the ellipse, *φ* represents the azimuth angle of the ellipse (i.e., the standing wave precession angle), and *β* represents the initial phase angle. Here, we mainly focus on the standing wave precession angle *φ.* Assuming that *Ω* ≪ *ω*, the trajectory of the point (*p*, *q*) is no longer a stationary ellipse. It is a precession ellipse, as shown in Figure 3b. However, the vibration period can still be approximated as *T* = 2π/*ω*.

The differential equations describing the standing wave precession angle *φ* are obtained using the method of averaging [27].

(22) $$\dot{\varphi }=-\frac{k_{1}}{2}\Omega ,$$

#### 2.3.2. Solution to the Non-Ideal Hemispherical Resonator

In order to facilitate the following analysis, the dynamic equation of the non-ideal hemispherical resonator is simplified into the following form.

(23) $$\left\{\begin{array}{l} \ddot{p}+\omega^{2}p=-k_{11}\Omega \dot{q}-k_{12}\Omega \dot{p}+(k_{13}\Omega^{2}-k_{14}\dot{\Omega }-k_{15}\Omega^{2}+\omega_{p})p+(-k_{16}\dot{\Omega }+k_{17}\Omega^{2}+k_{18}-k_{19}\Omega^{2})q,\\ \ddot{q}+\omega^{2}q=k_{21}\Omega \dot{p}+k_{22}\Omega \dot{q}+(k_{23}\Omega^{2}+k_{24}\dot{\Omega }-k_{25}\Omega^{2}+\omega_{q})q+(k_{26}\dot{\Omega }+k_{27}\Omega^{2}+k_{28}-k_{29}\Omega^{2})p, \end{array}\right.$$

where the coefficients or variables $\omega_{1}$, $\omega_{2}$, *k*_11_⋯*k*_19_ and *k*_21_⋯*k*_29_ are all constant values and the expressions for them are given as

(24) $$\begin{array}{l} k_{11}=\frac{1}{2}(c_{11}+c_{12}),\;\;\;k_{12}=\frac{1}{4}c_{15}(c_{21}+c_{22}),\;\;k_{13}=c_{13},\;\;k_{14}=\frac{1}{4}c_{15}c_{22},\;\;k_{15}=\frac{1}{4}c_{15}c_{24},\;\\ k_{16}=\frac{1}{2}c_{11},\;\;\;k_{17}=\frac{1}{2}c_{14},\;\;\;k_{18}=\frac{1}{2}c_{15}c_{26},\;\;\;k_{19}=\frac{1}{2}c_{15}c_{23},\;\;\;\omega_{1}^{2}=c_{16},\;\;\;\omega_{p}=\omega^{2}-\omega_{1}^{2},\\ k_{21}=\frac{1}{2}(c_{21}+c_{22}),\;\;\;k_{22}=\frac{1}{4}c_{25}(c_{11}+c_{12}),\;\;\;k_{23}=c_{23},\;\;k_{24}=\frac{1}{4}c_{25}c_{11},\;\;\;k_{25}=\frac{1}{4}c_{25}c_{14},\;\\ k_{26}=\frac{1}{2}c_{22},\;\;\;k_{27}=\frac{1}{2}c_{24},\;\;\;k_{28}=\frac{1}{2}c_{25}c_{16},\;\;\;k_{29}=\frac{1}{2}c_{25}c_{13},\;\;\;\omega_{2}^{2}=c_{26},\;\;\;\omega_{q}=\omega^{2}-\omega_{2}^{2}.\;\;\; \end{array}$$

The differential equations describing the standing wave precession angle *φ* are obtained using the method of averaging [27]

(25) $$\begin{array}{l} \dot{\varphi }=-\frac{1}{2\omega (a^{2}-b^{2})}[\sin\varphi \sin\varphi (a^{2}x_{5}-abx_{2}+abx_{3}-b^{2}x_{7})+\cos\varphi \cos\varphi (a^{2}x_{7}-abx_{3}+abx_{2}-b^{2}x_{5})\\ \;+\cos\varphi \sin\varphi (a^{2}x_{6}+a^{2}x_{8}+b^{2}x_{6}+b^{2}x_{8}+2abx_{4}+2abx_{1})], \end{array}$$

where the coefficients *x*_1_⋯*x*_8_ are all constant values and the expressions for them are given as

(26) $$\begin{array}{l} x_{1}=-k_{16}\dot{\Omega }+k_{17}\Omega^{2}+k_{18}-k_{19}\Omega^{2},\;\;\;x_{2}=k_{13}\Omega^{2}-k_{14}\dot{\Omega }-k_{15}\Omega^{2}+\omega_{p},\;\\ x_{3}=k_{23}\Omega^{2}+k_{24}\dot{\Omega }-k_{25}\Omega^{2}+\omega_{q},\;\;\;x_{4}=k_{26}\dot{\Omega }+k_{27}\Omega^{2}+k_{28}-k_{29}\Omega^{2},\;\\ x_{5}=k_{11}\omega \Omega ,\;\;\;x_{6}=k_{12}\omega \Omega ,\;\;\;x_{7}=k_{21}\omega \Omega ,\;\;\;x_{8}=k_{22}\omega \Omega \;. \end{array}$$

## 3. Results and Discussion

According to the above dynamic equations and the slow variable (standing wave precession angle *φ*) differential equations that describe the ideal and non-ideal hemispherical resonators, simulation experiments were carried out from two perspectives. One used the changing law of fast variables, the other used the changing law of slow variables.

### 3.1. Frequency Splitting and Angular Velocity

According to the geometric and physical parameters given in Table 1, the natural frequency of the ideal hemispherical resonator is *f* = 4956.1165 Hz, as shown in Equation (15), and the frequency splitting of the non-ideal hemispherical resonator is Δ*f* = 0.0397 Hz, as shown in Equation (19). 

The angular velocity of the hemispherical resonator is set to *Ω* = *C*(1 − *e*^−*ςt*^) [19], where the parameter *C* represents the magnitude of angular velocity. The larger *C* is, the larger the angular velocity is. The parameter *ς* represents the severity of the change in angular velocity. The larger the parameter *ς* is, the more drastic the changes in angular velocity are. Four angular velocities are set as shown in Table 2.

### 3.2. Comparison of the Change Law of Fast Variables

**Figure 4:**
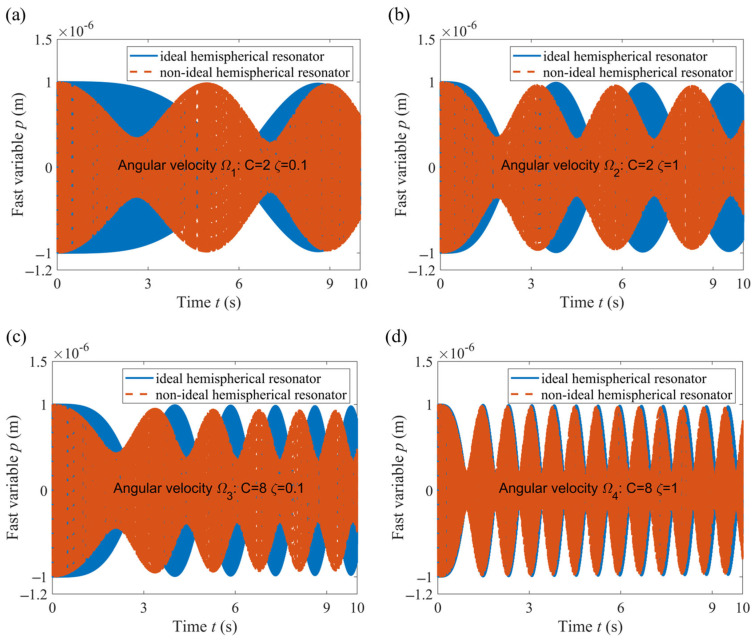
Fast variable *p*(*t*) changes over time. (**a**) Angular velocity *Ω*_1_; (**b**) Angular velocity *Ω*_2_; (**c**) Angular velocity *Ω*_3_; (**d**) Angular velocity *Ω*_4_.

The 4th order Runge–Kutta method is used to numerically solve the nonlinear Equations (14) and (16). The vibration of the hemispherical resonator is described by the fast variables *p*(*t*) and *q*(*t*), which indirectly reflect the precession of the standing wave. In response to the four angular velocities, the *p*(*t*) variables of the ideal and non-ideal hemispherical resonators are shown in Figure 4.

Comparing Figure 4a with Figure 4b, it was observed that as *ς* increases (in the case of *C* = 2), the *p*(*t*) of the non-ideal hemispherical resonator approaches the change law of the ideal hemispherical resonator. Similarly, comparing Figure 4c with Figure 4d, as *ς* increases (in the case of *C* = 8), the *p*(*t*) of the non-ideal hemispherical resonator approaches the change law of the ideal hemispherical resonator. Comparing Figure 4a with Figure 4c, it was observed that as *C* increases (in the case of *ς* = 0.1), the *p*(*t*) of the non-ideal hemispherical resonator approaches the change law of the ideal hemispherical resonator. Similarly, comparing Figure 4b with Figure 4d, as *C* increases (in the case of *ς* = 1), the *p*(*t*) of the non-ideal hemispherical resonator approaches the change law of the ideal hemispherical resonator.

**Figure 5:**
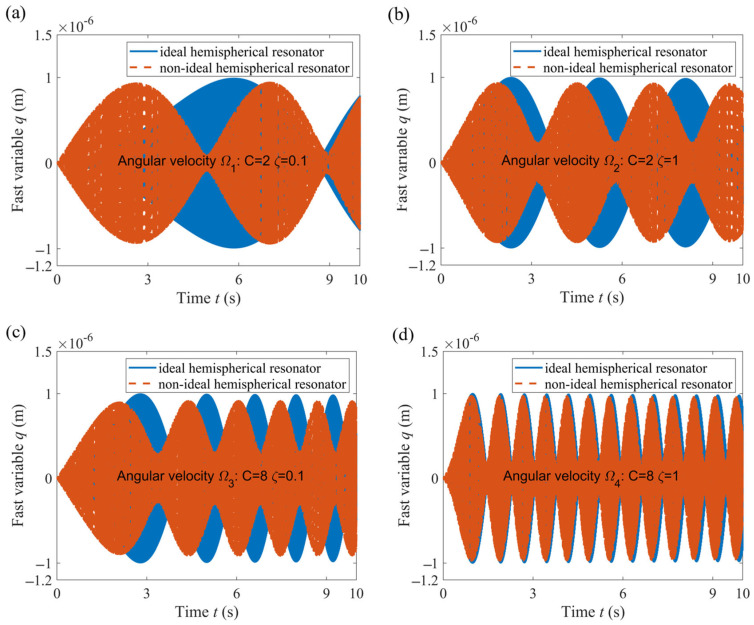
Fast variable *q*(*t*) changes with time. (**a**) Angular velocity *Ω*_1_; (**b**) Angular velocity *Ω*_2_; (**c**) Angular velocity *Ω*_3_; (**d**) Angular velocity *Ω*_4_.

In response to the four angular velocities, the *q*(*t*) values of the ideal and non-ideal hemispherical resonators are shown in Figure 5. Comparing Figure 5a with Figure 5b, it was observed that as *ς* increases (in the case of *C* = 2), the *q*(*t*) of the non-ideal hemispherical resonator approaches the change law of the ideal hemispherical resonator. Similarly, comparing Figure 5c with Figure 5d, it was observed that as *ς* increases (in the case of *C* = 8), the *q*(*t*) of the non-ideal hemispherical resonator approaches the change law of the ideal hemispherical resonator. Comparing Figure 5a with Figure 5c, it was observed that as *C* increases (in the case of *ς* = 0.1), the *q*(*t*) of the non-ideal hemispherical resonator approaches the change law of the ideal hemispherical resonator. Similarly, comparing Figure 5b with Figure 5d, it was observed that as *C* increases (in the case of *ς* = 1), the *q*(*t*) of the non-ideal hemispherical resonator approaches the change law of the ideal hemispherical resonator.

Compared with the results of references [19,20], which studied the dynamics of an elastic ring resonator under high-intensity dynamic processes in the presence of mass defects, the influence of high-intensity dynamic processes on the fast variables (generalized displacement) of the hemispherical resonator is similar to that observed on the ring resonator. Due to the fact that the standing waves are formed by the superposition of two modes, the results indirectly prove that taking into account the nonlinear terms about the square of the angular velocity and the angular acceleration in the dynamic equation can weaken the influence of the 4th harmonic component of the mass defect on standing wave drift.

### 3.3. Comparison of the Change Law of Slow Variable

In order to highlight the influence of high dynamics on the standing wave of a hemispherical resonator with a mass defect, simulation experiments were conducted under conditions of no angular velocity, a small uniform angular velocity and high dynamic, respectively.

#### 3.3.1. No Angular Velocity and Small Uniform Angular Velocity

In order to observe the influence of a mass defect on the vibration performance of the non-ideal hemispherical resonator, the change of the slow variable *φ* (standing wave precession angle) between the ideal resonator and non-ideal resonator were compared according to Equations (22) and (25). The word “ignored” indicates that we disregarded the nonlinear terms about the square of angular velocity and the angular acceleration in Equations (22) and (25).

**Figure 6:**
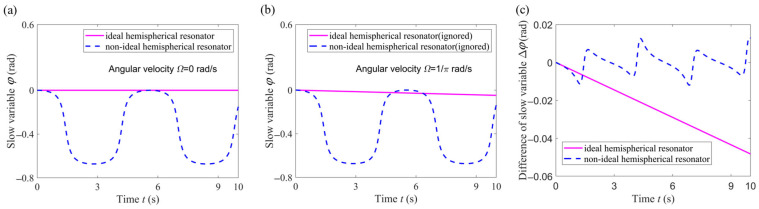
Change law of slow variable *φ*. (**a**) No angular velocity; (**b**) Small uniform angular velocity. (**c**) Difference of slow variable Δ*φ* under two different angular velocities.

The standing wave precession angle serves as a bridge used by the hemispherical resonator gyroscope to reflect the angular velocity, which directly affects the sensitivity of the gyroscope. From Figure 6a, it can be seen that in the case of no angular velocity, in contrast to the performance of an ideal resonator, a mass defect causes periodic fluctuations in the standing wave precession angle *φ*. From Figure 6b, it can be seen that the mass defect causes nonlinear changes in the standing wave precession angle *φ*, exhibiting periodic fluctuations (the ideal resonator changes linearly). From Figure 6c, it can be seen that the difference of slow variable Δ*φ*, under two different angular velocities, exhibits a monotonically decreasing trend over time in the ideal resonator. However, this difference shows a periodic fluctuating trend over time in the non-ideal resonator. This indicates that a mass defect of a resonator can cause the standing wave to oscillate periodically over time. Hence, when subjected to a small uniform angular velocity rotation, the influence of a mass defect on the standing wave precession angle is significant. This is why researchers compensate for standing wave drift in a hemispherical resonator gyroscope control system. 

#### 3.3.2. High-Intensity Dynamic

To highlight the impact of high dynamics on the standing wave precession angle, for these four angular velocities, the changes in the slow variable *φ* of the ideal hemispherical resonator, the non-ideal hemispherical resonator, the ideal resonator hemispherical resonator (ignored) and the non-ideal hemispherical resonator (ignored) are shown in the figures below. The word “ignored” indicates that we disregarded the nonlinear terms about the square of angular velocity and the angular acceleration in Equations (22) and (25).

**Figure 7:**
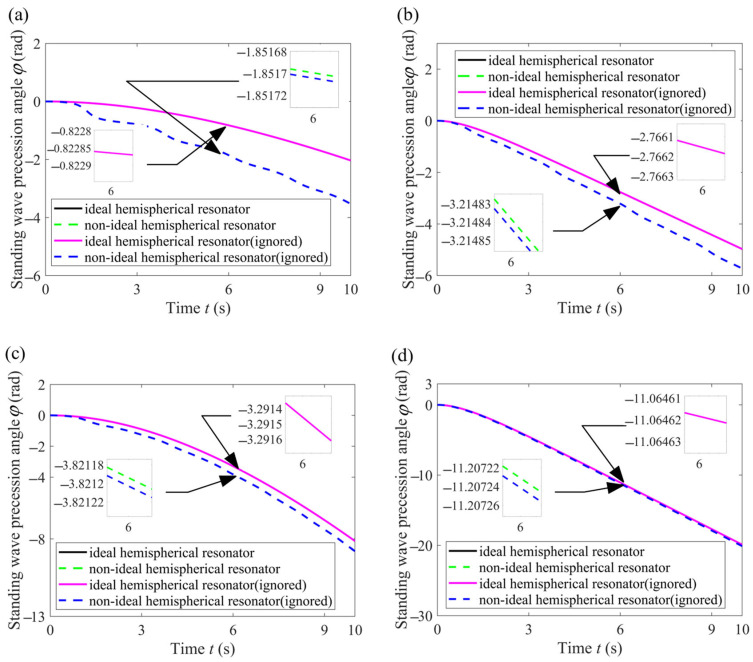
Change law of the standing wave precession angle *φ*. (**a**) Angular velocity *Ω*_1_; (**b**) Angular velocity *Ω*_2_; (**c**) Angular velocity *Ω*_3_; (**d**) Angular velocity *Ω*_4_.

On the whole, ignoring the nonlinear terms about the square of the angular velocity and the angular acceleration causes less error in modeling both the ideal and the non-ideal resonators. Comparing Figure 7a with Figure 7b, it was observed that as *ς* increases (in the case of *C* = 2), the change law of the non-ideal hemispherical resonator (ignored) approaches that of the ideal hemispherical resonator (ignored). Similarly, comparing Figure 7c to Figure 7d, it was observed that as *ς* increases (in the case of *C* = 8), the change law of the non-ideal hemispherical resonator (ignored) approaches that of the ideal hemispherical resonator (ignored). Comparing Figure 7a with Figure 7c, it was observed that as *C* increases (in the case of *ς* = 0.1), the change law of the non-ideal hemispherical resonator (ignored) approaches that of the ideal hemispherical resonator (ignored). Similarly, comparing Figure 7b with Figure 7d, it was observed that as *C* increases (in the case of *ς* = 1), the change law of the non-ideal hemispherical resonator (ignored) approaches that of the ideal hemispherical resonator (ignored).

In order to observe more clearly the influence of ignoring these nonlinear terms on the standing waves of ideal and non-ideal hemispherical resonators, respectively, we adopted Δ*φ* (the difference in standing wave precession angle) as an evaluation indicator.

(27) $$\Delta \varphi =\varphi_{\mathrm{non-ignore}}-\varphi_{\mathrm{ignore}}$$

where subscript “ignore and non-ignore” represent ignoring and not ignoring these nonlinear terms in the dynamic equations, respectively. For each group (Ideal resonator group or Non-ideal resonator group), the evaluation indicator Δ*φ* is simulated under these four angular velocities, as shown in the following figures.

**Figure 8:**
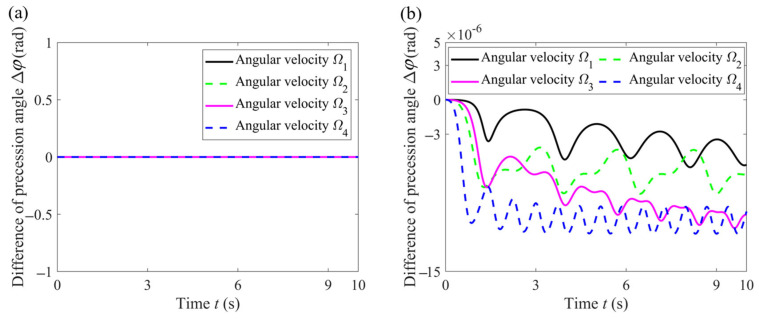
Δ*φ* in each group. (**a**) Ideal resonator group. (**b**) Non-ideal resonator group.

As can be seen from Figure 8a, ignoring the nonlinear terms affecting the square of the angular velocity and the angular acceleration results in zero error for the ideal resonator, which is also consistent with the results of Equation (22). From Figure 8b, it can be seen that for a non-ideal resonator, the difference in the standing wave precession angle caused by ignoring the nonlinear terms becomes more and more significant as the angular velocity changes more and more sharply. It is important to note that the standing wave precession angle is sensitive to these nonlinear terms.

In addition to comparing the standing wave precession of ideal and non-ideal hemispherical resonators under high-intensity dynamics, the next step is to study the influence of different mass defects on the standing wave precession of non-ideal hemispherical resonators under the same high-intensity dynamics. The mass defects cause the standing wave precession angle of the hemispherical resonator to drift. Meanwhile, in order to observe more clearly the weakening effect of high-intensity dynamic processes on this phenomenon, we adopted *λ* (the ratio of the standing wave precession angle of the hemispherical resonators) as an evaluation indicator.

(28) $$\lambda =\frac{\varphi_{\mathrm{non-ideal}}}{\varphi_{\mathrm{ideal}}}$$

where the subscript “ideal and non-ideal” represent ideal hemispherical resonator without mass defects and non-ideal hemispherical resonator with mass defects, respectively. For different mass defects (small or large), *λ* was simulated under these four angular velocities, as shown in the following figures.

Hemispherical resonators are usually made of fused quartz shaped by precise mechanical processing, and their mass defects are relatively small, often resulting in frequency splitting of less than 0.1 Hz. According to Equation (11), the dimension of mass defects is reflected by parameter *ε*_4_, the relative amplitude of the 4th harmonic of density anisotropy, which is set to two cases, as shown in Table 3. The other parameters’ settings remain unchanged, as shown in Table 1, and the frequency splitting is shown in Table 3, calculated by Equation (19). 

**Figure 9:**
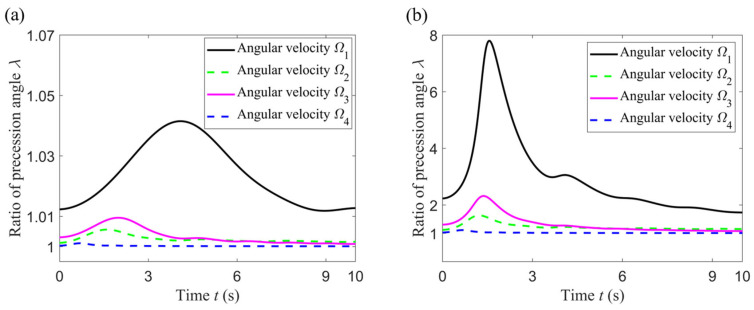
Ratio of standing wave precession angle of the hemispherical resonators. (**a**) In the case of a small mass defect; (**b**) In the case of a large mass defect.

According to Figure 9 and Table 2, it can be seen that for the same angular velocity, the larger the mass defect, the larger the ratio of precession angle *λ*. For the same mass defect, comparing angular velocity *Ω*_1_ with angular velocity *Ω*_3_ or comparing angular velocity *Ω*_2_ with angular velocity *Ω*_4_, it can be seen that as *C* increases, the value of *λ* decreases, which reflects the fact that the larger the angular velocity amplitude, the smaller the value of *λ*. Furthermore, for the same mass defect, comparing angular velocity *Ω*_1_ with angular velocity *Ω*_2_ or comparing angular velocity *Ω*_3_ with angular velocity *Ω*_4_, it can be seen that as *ς* increases, the value of *λ* decreases, which reflects that the more drastic the change in angular velocity, the smaller the value of *λ*. The closer the value of *λ* is to 1, the closer the standing wave precession angle of non-ideal resonator is to that of the ideal resonator. In other words, the value of *λ* reflects the extent of drift of the standing wave precession angle. The smaller the value of *λ*, the weaker the extent of drift, that is to say, the weaker the influence of mass defects. From the above we can determine that a high-intensity dynamic process can weaken the influence of a mass defect on the standing wave drift of a non-ideal hemispherical resonator. The extent of this weakening effect varies with the dimensions of the mass defect.

## 4. Conclusions

In this paper, based on the thin shell theory of elasticity, the nonlinear terms about the square of the angular velocity and the angular acceleration, as well as the mass defects of the hemispherical resonator are taken into account. A dynamic model of the hemispherical resonator is established precisely by the Lagrange’s second kind equation. The dynamic model can be equivalently transformed into a point mass harmonic vibration model in a two-dimensional space, and it can also be analyzed using a method of averaging that separates the slow variables from the fast variables in the established model. The simulation experiments were carried out from two perspectives, one using the change law of the fast variables, the other using the change law of the slow variable. The results show that mass defects will cause the standing wave of a resonator to drift, even if the hemispherical resonator is in a non-rotating state. In addition, the larger the mass defects, the greater their influence on the standing wave drift of a non-ideal hemispherical resonator/* under the same high-intensity dynamics.

Whether from the view of the fast variables or the slow variables, the larger the angular velocity amplitude is, or the more drastically the angular velocity changes, the more closely the changing law of the non-ideal hemispherical resonator approaches that of the ideal hemispherical resonator, which reveals that the high-intensity dynamic process can weaken the influence of the 4th harmonic component of the mass defects on the standing wave drift of a non-ideal hemispherical resonator. The extent of this weakening effect varies with the dimensions of the mass defects. The research in this paper provides important guidance for improving gyroscope accuracy in the future by dynamically compensating for the drift of an unbalanced hemispherical resonator in an environment of large angular rate and angular acceleration, particularly in the case of high frequency vibrations. 

To further improve the accuracy of hemispherical resonator gyroscopes, a balancing process (such as mechanical balancing, chemical balancing, laser balancing or ion beam balancing) must be applied during resonator manufacturing to reduce frequency splitting caused by mass defects. In addition, in order to avoid damaging hemispherical resonators, non-contact testing should be used to identify mass defects in them, such as detecting vibration signals from the lips of resonators using a Doppler laser vibrometer and measuring electrical signals using electrostatic excitation and detection, etc.

## Figures and Tables

**Table 1:** Geometric and physical parameters of the hemispherical resonator.

Symbol	Variable	Value [unit]
*R*	Radius of the middle surface	15 mm
*H*	Thickness	0.85 mm
*E*	Young’s modulus	76.7 GPa
*ρ*	Density	2200 kg/m^3^
*μ*	Poisson’s ratio	0.17
*ρ*_0_	Average density	2200 kg/m^3^
*ε*_4_	Relative amplitude	1.0 × 10^−4^
*θ*_4_	Relative phase	π/7 rad

**Table 2:** Angular velocity.

Symbol	Parameter *C* (rad/s)	Parameter *ς* (1/s)
Angular velocity *Ω*_1_	2.0	0.1
Angular velocity *Ω*_2_	2.0	1.0
Angular velocity *Ω*_3_	8.0	0.1
Angular velocity *Ω*_4_	8.0	1.0

**Table 3:** Dimension of mass defects.

Case Number	Parameter *ε*_4_	Frequency Splitting Δ*f* (Hz)
Case 1 (small)	1.0 × 10^−5^	Δ*f*_1_ = 0.00397
Case 2 (large)	1.0 × 10^−4^	Δ*f*_2_ = 0.03970

## Data Availability

Data are available from the corresponding author upon reasonable request.

## Appendix A. Coefficients of Strain Energy and Kinetic Energy

The expression for coefficient *b*_0_ of Equation (5) can be given as

(A1) $$b_{0}=\frac{Eh^{3}\pi }{24(1-\mu^{2})}\int_{0}^{\frac{\pi }{2}}\sin\alpha \left\{\begin{array}{l} \frac{1}{R^{2}}{\left[(1-\frac{4}{\sin^{2}\alpha })W(\alpha )+\frac{\partial W(\alpha )}{\partial \alpha }\cot\alpha \right]}^{2}+\frac{8(1-\mu )}{R^{2}}{\left[\frac{\partial W(\alpha )}{\partial \alpha }-W(\alpha )\cot\alpha \right]}^{2}\\ +\frac{2\mu }{R^{2}}{\left[W(\alpha )+\frac{\partial^{2}W(\alpha )}{\partial \alpha^{2}}\right]}^{2}\left[(1-\frac{4}{\sin^{2}\alpha })W(\alpha )+\frac{\partial W(\alpha )}{\partial \alpha }\cot\alpha \right]\frac{1}{R^{2}}{\left[W(\alpha )+\frac{\partial^{2}W(\alpha )}{\partial \alpha^{2}}\right]}^{2} \end{array}\right\}d\alpha$$

The expressions for coefficients *a*_0_, *a*_1_, *a*_2_ and *a*_3_ of Equation (10) can be given as

(A2) $$\begin{array}{l} a_{0}=\frac{\pi h}{2}\int_{0}^{\frac{\pi }{2}}2\rho R^{4}\sin^{3}\alpha d\alpha \\ a_{1}=\frac{\pi hR^{2}}{2}\int_{0}^{\frac{\pi }{2}}\rho \left[U^{2}(\alpha )+V^{2}(\alpha )+W^{2}(\alpha )\right]\sin\alpha d\alpha \\ a_{2}=\pi hR^{2}\int_{0}^{\frac{\pi }{2}}\rho V(\alpha )\left[U(\alpha )\cos\alpha +W(\alpha )\sin\alpha \right]\sin\alpha d\alpha \\ a_{3}=\frac{\pi hR^{2}}{2}\int_{0}^{\frac{\pi }{2}}\rho \left[U^{2}(\alpha )\cos^{2}\alpha +2U(\alpha )V(\alpha )\sin\alpha \cos\alpha +V^{2}(\alpha )+W^{2}(\alpha )\sin^{2}\alpha \right]\sin\alpha d\alpha \end{array}$$

The expressions for coefficients ${\bar{a}}_{0}\cdots {\bar{a}}_{8}$ of Equation (12) can be given as

(A3) $$\begin{array}{l} {\bar{a}}_{0}=\frac{71h\rho_{0}}{2712}(80R^{4}+40R^{2}h^{2}+h^{4})\\ {\bar{a}}_{1}=\frac{1}{R^{2}}[\frac{71}{10848}h\rho_{0}(\frac{17254}{47}R^{4}+\frac{3920}{107}h^{4}+\frac{42467}{94}R^{2}h^{2}-\frac{3268}{985}h^{4}\varepsilon_{4}\cos(4\theta_{4})\\ \;\;+\frac{5043}{38}R^{4}\varepsilon_{4}\cos(4\theta_{4})-\frac{3848}{197}R^{2}h^{2}\varepsilon_{4}\cos(4\theta_{4}))]\\ {\bar{a}}_{2}=\frac{1}{R^{2}}[\frac{71}{10848}h\rho_{0}(\frac{17254}{47}R^{4}+\frac{3920}{107}h^{4}+\frac{42467}{94}R^{2}h^{2}+\frac{3268}{985}h^{4}\varepsilon_{4}\cos(4\theta_{4})\\ \;\;-\frac{5043}{38}R^{4}\varepsilon_{4}\cos(4\theta_{4})+\frac{3848}{197}R^{2}h^{2}\varepsilon_{4}\cos(4\theta_{4}))]\\ {\bar{a}}_{3}=\frac{1}{R^{2}}[\frac{71}{5254}h\rho_{0}(\frac{16963}{33}R^{4}+160R^{6}+\frac{4102}{197}h^{4}+\frac{91891}{192}R^{2}h^{2}+2R^{2}h^{4}+80R^{4}h^{2}\\ \;\;+\frac{3268}{985}h^{4}\varepsilon_{4}\cos(4\theta_{4})+\frac{2635}{94}R^{4}\varepsilon_{4}\cos(4\theta_{4})+\frac{10430}{197}R^{2}h^{2}\varepsilon_{4}\cos(4\theta_{4}))]\\ {\bar{a}}_{4}=\frac{1}{R^{2}}[\frac{8272}{589}h\rho_{0}(\frac{15404}{85}+34R^{4}+34R^{6}+33h^{4}+34R^{2}h^{2}+\frac{261}{99}\times 10^{32}R^{2}h^{4}+34R^{4}h^{2}\\ \;\;-\frac{217}{50}h^{4}\varepsilon_{4}\cos(4\theta_{4})-\frac{16588}{449}R^{4}\varepsilon_{4}\cos(4\theta_{4})-\frac{5475}{79}R^{2}h^{2}\varepsilon_{4}\cos(4\theta_{4}))]\\ {\bar{a}}_{5}=\frac{1}{R^{2}}[\frac{71}{10848}h\rho_{0}(\frac{11948}{47}R^{4}+320R^{6}+\frac{2500}{107}h^{4}+240R^{2}h^{2}+4R^{2}h^{4}+160R^{4}h^{2}\\ \;\;-\frac{3623}{364}h^{4}\varepsilon_{4}\cos(4\theta_{4})+\frac{5391}{72}R^{4}\varepsilon_{4}\cos(4\theta_{4})-\frac{27091}{216}R^{2}h^{2}\varepsilon_{4}\cos(4\theta_{4}))]\\ {\bar{a}}_{6}=\frac{1}{R^{2}}[\frac{71}{10848}h\rho_{0}(\frac{11948}{47}R^{4}+320R^{6}+\frac{2500}{107}h^{4}+240R^{2}h^{2}+4R^{2}h^{4}+160R^{4}h^{2}\\ \;\;+\frac{3623}{364}h^{4}\varepsilon_{4}\cos(4\theta_{4})-\frac{5391}{72}R^{4}\varepsilon_{4}\cos(4\theta_{4})+\frac{27091}{216}R^{2}h^{2}\varepsilon_{4}\cos(4\theta_{4}))]\\ {\bar{a}}_{7}=\frac{1}{R^{2}}[\frac{71}{5424}h\rho_{0}(\frac{11948}{47}R^{4}+160R^{6}+\frac{2500}{107}h^{4}+240R^{2}h^{2}+2R^{2}h^{4}+80R^{4}h^{2}\\ \;\;-\frac{3623}{364}h^{4}\varepsilon_{4}\cos(4\theta_{4})+\frac{5391}{72}R^{4}\varepsilon_{4}\cos(4\theta_{4})-\frac{27091}{216}R^{2}h^{2}\varepsilon_{4}\cos(4\theta_{4}))]\\ {\bar{a}}_{8}=-\frac{1}{R^{2}}[h\rho_{0}\varepsilon_{4}\sin(4\theta_{4})\;(34R^{4}+33R^{2}h^{2}+\frac{89}{20493}h^{4}+\frac{3623}{442})] \end{array}$$
